# Efficient and accurate tobacco leaf maturity detection: an improved YOLOv10 model with DCNv3 and efficient local attention integration

**DOI:** 10.3389/fpls.2024.1474207

**Published:** 2025-01-03

**Authors:** Yi Shi, Hong Wang, Fei Wang, Yingkuan Wang, Jianjun Liu, Long Zhao, Hui Wang, Feng Zhang, Qiongmin Cheng, Shunhao Qing

**Affiliations:** ^1^ College of Agricultural Equipment Engineering, Henan University of Science and Technology, Luoyang, Henan, China; ^2^ Henan Province Tobacco Company, Luoyang Company, Luoyang, China; ^3^ Academy of Agricultural Planning and Engineering, Ministry of Agriculture and Rural Affairs, Beijing, China; ^4^ Henan Province Tobacco Company, Zhengzhou, China; ^5^ College of Horticulture and Plant Protection, Henan University of Science and Technology, Luoyang, China

**Keywords:** tobacco leaf maturity, YOLOv10, DCNv3, efficient local attention, targeted detection

## Abstract

The precise determination of tobacco leaf maturity is pivotal for safeguarding the taste and quality of tobacco products, augmenting the financial gains of tobacco growers, and propelling the industry’s sustainable progression. This research addresses the inherent subjectivity and variability in conventional maturity evaluation techniques reliant on human expertise by introducing an innovative YOLOv10-based method for tobacco leaf maturity detection. This technique facilitates a rapid and non-invasive assessment of leaf maturity, significantly elevating the accuracy and efficiency of tobacco leaf quality evaluation. In our study, we have advanced the YOLOv10 framework by integrating DCNv3 with C2f to construct an enhanced neck network, designated as C2f-DCNv3. This integration is designed to augment the model’s capability for feature integration, particularly concerning the morphological and edge characteristics of tobacco leaves. Furthermore, the incorporation of the Efficient Local Attention (ELA) mechanism at multiple stages of the model has substantially enhanced the efficiency and fidelity of feature extraction. The empirical results underscore the model’s pronounced enhancement in performance across all maturity classifications. Notably, the overall precision (P) has been elevated from 0.939 to 0.973, the recall rate (R) has improved from 0.968 to 0.984, the mean average precision at 50% intersection over union (mAP50) has advanced from 0.984 to 0.994, and the mean average precision across the 50% to 95% intersection over union range (mAP50-95) has risen from 0.962 to 0.973. This research presents the tobacco industry with a novel rapid detection instrument for tobacco leaf maturity, endowed with substantial practical utility and broad prospects for application. Future research endeavors will be directed towards further optimization of the model’s architecture to bolster its generalizability and to explore its implementation within the realm of actual tobacco cultivation and processing.

## 1 Introduction

The maturity of tobacco leaves is a critical factor that directly influences their quality and, consequently, the taste and value of tobacco products (Cai et al., 2005; Yin et al., 2019). This is of paramount importance for the sustainability of the tobacco industry and the economic well-being of tobacco farmers (Kays, 2011). Achieving consistent and accurate assessments of tobacco leaf maturity is vital, as it enables more precise harvesting and curing methods that optimize both the aromatic profile and minimize harmful chemicals in the leaves (Cakir and Cebi, 2010). Traditionally, farmers have relied on subjective experience to assess leaf maturity, which can lead to inconsistent outcomes and missed opportunities for optimal harvest timing (Chen et al., 2023; Sun et al., 2023b).

Despite the progress in tobacco classification techniques, including the use of hyperspectral imaging and machine learning models, the practical adoption of these methods has been limited due to high equipment costs, complexity, and the need for specialized skills (Chen et al., 2021). These factors highlight a significant technical gap: the need for an accessible, non-destructive method for assessing tobacco leaf maturity in the field.

The advantage of object detection methods in maturity recognition lies in their ability to accurately localize and categorize each target within images, thereby enabling rapid and efficient identification and classification of agricultural products at various stages of ripeness. To meet the practical needs of farmers, our research proposes an innovative solution by leveraging advances in machine vision and object detection for real-time, accurate, and affordable field-based maturity detection of tobacco leaves. Specifically, we develop a lightweight YOLOv10-based algorithm integrated with Deformable Convolutional Networks (DCNv3) and an Enhanced Lightweight Attention (ELA) mechanism. Our approach emphasizes real-time processing, affordability, and accuracy, addressing the challenges in field conditions. The primary contributions of this study are as follows:

1. We propose an advanced network structure combining YOLOv10 and DCNv3, enhancing feature aggregation and detection accuracy.
2. We introduce the ELA attention mechanism to replace the PSA module in the YOLOv10 backbone, improving feature representation.
3. We incorporate the ELA attention mechanism between the backbone and neck networks, further boosting overall model performance.
4. We conduct comprehensive experiments analyzing the influence of various network architectures and attention mechanisms on detection efficacy, aiming to optimize the lightweight performance of the model.

The remainder of this paper is organized as follows: Section 2 presents a detailed literature review of recent advancements in tobacco leaf classification and detection technologies. Section 3 describes our proposed method, including the YOLOv10 architecture and the ELA attention mechanism. Section 4 provides the experimental setup and results. Finally, Section 5 concludes the paper and outlines potential directions for future work.

## 2 Related work

In recent years, significant advances have been made in the use of spectral data and machine learning for the detection and classification of tobacco leaves. These technologies have proven effective in determining the maturity and quality of leaves, though challenges such as high costs and complex implementations remain.

Spectral imaging has emerged as a powerful tool for the classification of agricultural products, including tobacco leaves. Early efforts, such as those by Long et al. (2019), utilized hyperspectral imaging combined with Savitzky-Golay smoothing filters and multiplicative scatter correction, achieving an impressive 99% classification accuracy of tobacco leaves and impurities. Similarly, Lu et al. (2023) refined the maturity assessment of flue-cured tobacco using Partial Least Squares Discriminant Analysis (PLS-DA), obtaining 99.32% accuracy on the validation set.

However, despite their high accuracy, these hyperspectral approaches face notable barriers, including the cost of spectrometers and their limited portability, making them less accessible to the average tobacco farmer. The reliance on specialized technical skills further complicates the wide adoption of such methods in practical farming scenarios (Beć et al., 2021; Hussain et al., 2018).

In response to the limitations of hyperspectral imaging, machine learning models have been increasingly applied to tobacco leaf classification and detection (Zhang et al., 2024). Li et al. (2021) designed a lightweight network based on MobileNetV2 for assessing tobacco leaf maturity. This model balanced accuracy with computational efficiency, making it more practical for real-world deployment. Similarly, Jia et al. (2023) proposed a model based on YOLOv7 and the LWC algorithm for detecting mixed tobacco strands. This model achieved a high detection accuracy (mAP@0.5 = 0.932) and fast processing speed, demonstrating the viability of real-time detection in agriculture. Xiong et al. (2024) introduced the DiffuCNN model, designed for detecting tobacco diseases in complex, low-resolution environments. This model incorporated a diffusion enhancement module and achieved a precision of 0.98 with a processing speed of 62 FPS, outperforming other models in accuracy and efficiency. Meanwhile, He et al. (2023) developed the FSWPNet model, combining pyramid feature fusion with shifted window self-attention for improved classification of tobacco leaves, achieving an average classification precision of 75.8%.

Deep learning models, particularly those based on convolutional neural networks (CNNs), have played a significant role in advancing agricultural object detection (Biradar and Hosalli, 2024; Kang and Chen, 2020; LeCun et al., 2015; Zhao et al., 2022). The You Only Look Once (YOLO) series (Hussain, 2023) and SSD (Liu et al., 2016) exemplify single-stage algorithms, which swiftly localize and classify objects in a unified forward pass, aligning with the needs of real-time detection tasks (Soviany and Ionescu, 2018). Conversely, two-stage algorithms, such as Faster R-CNN (Ren et al., 2016) and Sparse R-CNN (Sun et al., 2023a), initiate with a Region Proposal Network (RPN) to delineate potential object regions, proceeding with classifiers for nuanced classification and localization (Du et al., 2020; Zhang et al., 2023a, 2021). Single-stage algorithms excel in their rapid and efficient processing, well-suited for high-speed application contexts (He et al., 2024). The YOLO series of models, such as YOLOv5, YOLOv6, and YOLOv7, have demonstrated their suitability for real-time detection tasks due to their single-stage nature, which allows for rapid localization and classification (Hussain, 2023). Although two-stage algorithms like Faster R-CNN offer higher precision, single-stage models are better suited for real-time applications due to their speed and reduced computational requirements (Bacea and Oniga, 2023).

Despite these advances, most research has focused on post-harvest tobacco leaf classification, a destructive process that may lead to waste. Few studies have explored non-destructive, field-based methods for detecting tobacco leaf maturity. This represents a critical gap in the literature, as non-destructive methods would allow for more accurate and timely harvesting decisions, ultimately benefiting both the quality of the tobacco and the economic returns for farmers (Zhang et al., 2023b).

Furthermore, the integration of attention mechanisms and deformable convolutions has been limited in the context of tobacco leaf detection. Recent studies have demonstrated the potential of these techniques to improve feature extraction and enhance model performance (Cheng et al., 2024; Du et al., 2025; Qing et al., 2024), suggesting that their incorporation into lightweight models like YOLOv10 could address both the accuracy and efficiency needs of practical agricultural applications.

The existing literature highlights several successful applications of spectral imaging and deep learning in tobacco leaf classification. However, the technical challenges associated with hyperspectral imaging and the lack of non-destructive methods for assessing tobacco leaf maturity underscore the need for new approaches. Our research builds upon these prior studies by introducing a YOLOv10-based lightweight model that incorporates DCNv3 and the ELA attention mechanism, addressing both the accuracy and computational constraints of field-based tobacco leaf maturity detection.

## 3 Materials and methods

### 3.1 Data collection and dataset construction

**Figure 1:**
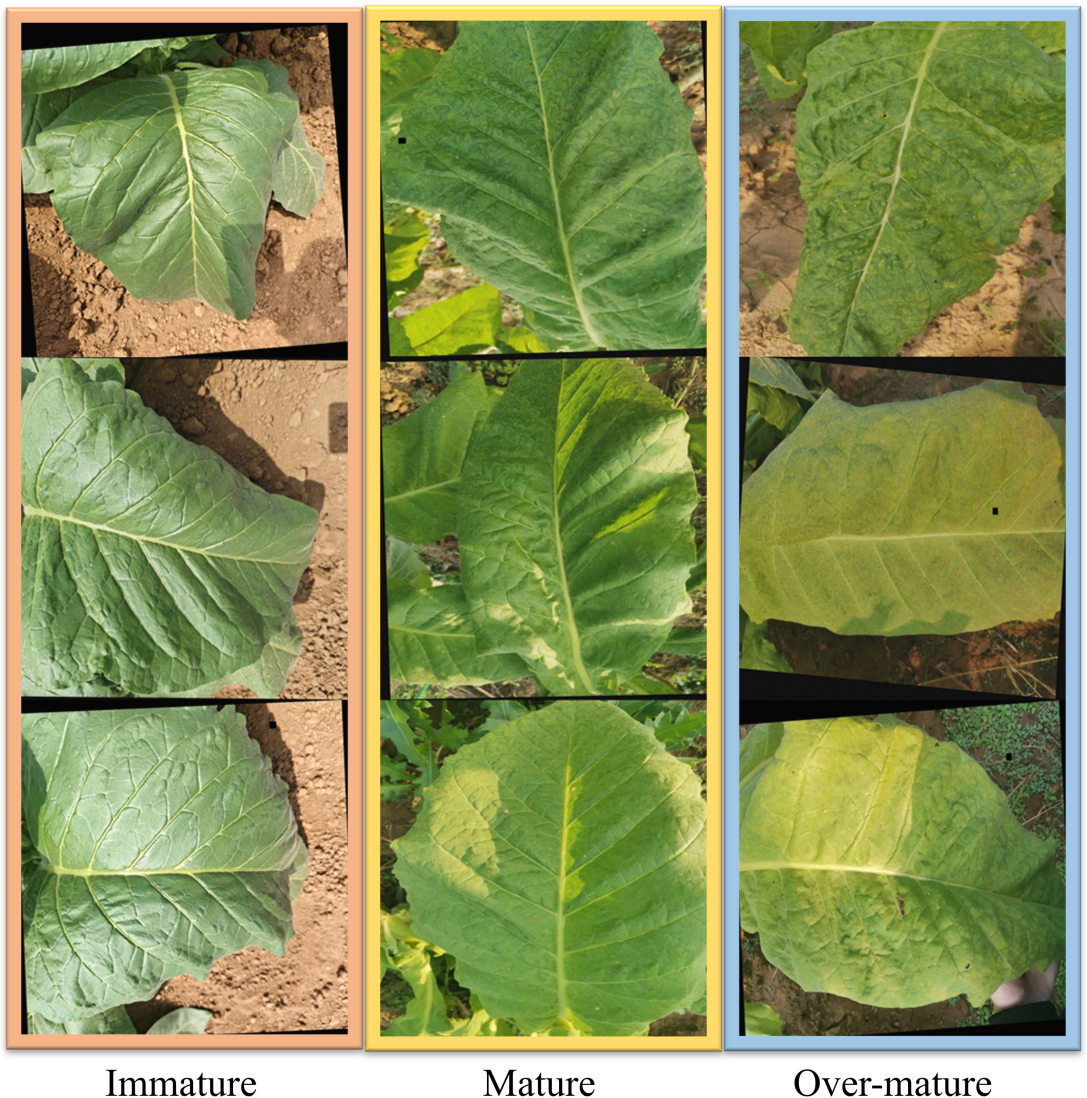
The sample image of the tobacco maturity dataset.

The research utilized a dataset of tobacco leaf maturity images, which was established from the collection of leaves in the tobacco cultivation region of Luoning County, Luoyang City, within Henan Province. For the acquisition of field data, the study employed the rear camera of a Huawei Honor 20 smartphone, featuring a 32-megapixel high-resolution sensor. To minimize the impact of lighting conditions on the leaf maturity recognition, the data was collected exclusively during daylight and under clear skies. To further augment the complexity of the dataset and enhance the robustness of our model, we employed data augmentation techniques such as rotation, scaling, flipping, and the addition of noise. The tobacco leaves were classified into three distinct maturity stages: immature, mature, and over-mature. Immature leaves, characterized by their green color, are not harvest-ready. Mature leaves are identified as the optimal stage for harvesting without compromising the final product’s quality. Over-mature leaves, indicative of an excessive degree of maturity, are prone to significant losses during the harvesting and subsequent processing stages. In this study, the dataset was randomly partitioned following an 8:1:1 ratio into training, validation, and test sets, respectively. The training set consists of 1,752 images, the validation set contains 370 images, and the test set comprises 373 images. Representative images from the developed tobacco leaf maturity dataset are depicted in **Figure 1**.

### 3.2 Constructing the tobacco maturity detection model

#### 3.2.1 The basic network structure of YOLOv10n

**Figure 2:**
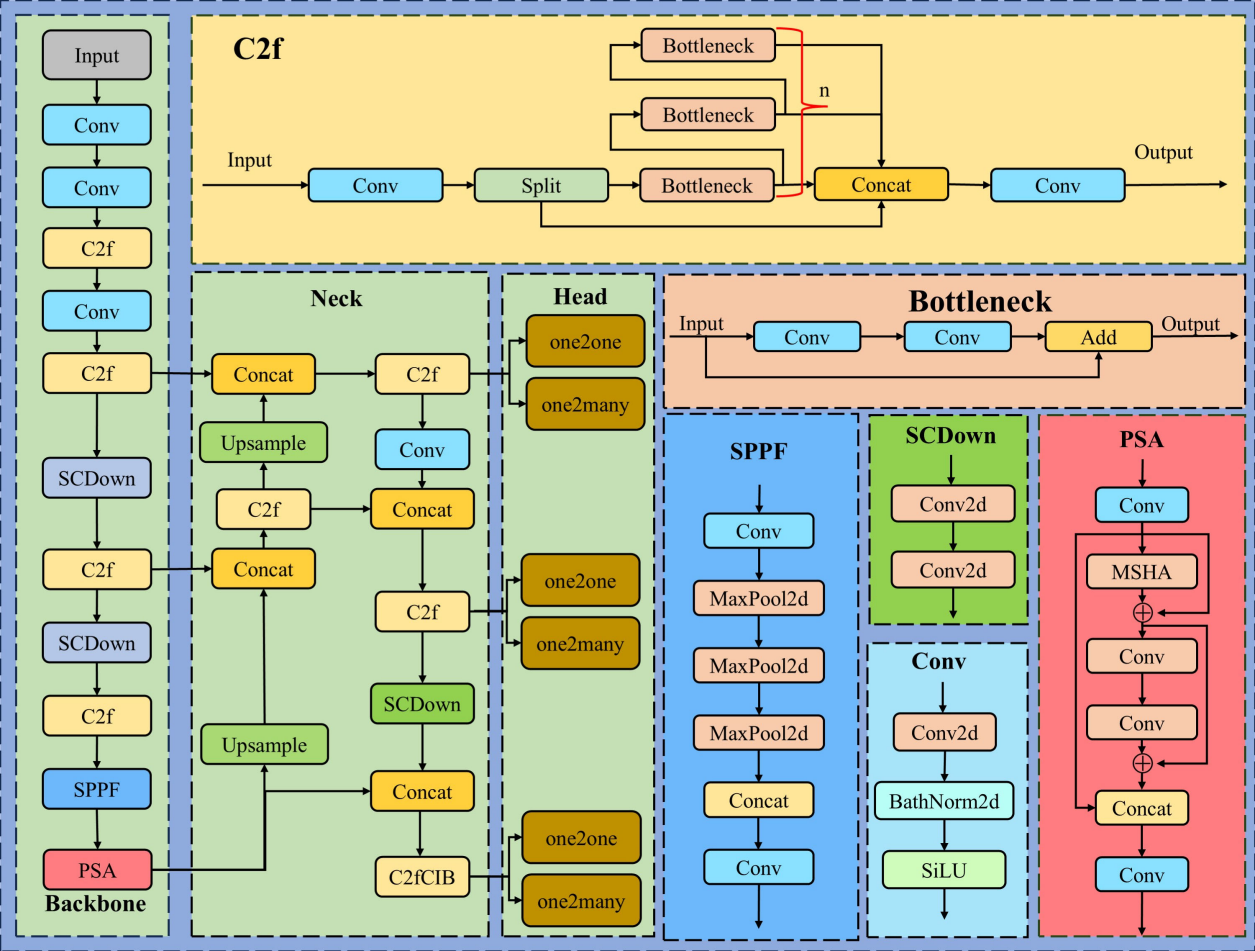
The structure of YOLOv10.

YOLOv10, the state-of-the-art real-time, end-to-end object detection model from the research team at Tsinghua University (Wang et al., 2024), stands as the pinnacle of the YOLO series. It preserves the real-time detection performance while substantially increasing the accuracy and efficiency of detection through a series of innovative advancements. The principal network framework is elegantly portrayed in **Figure 2**.

YOLOv10 has discarded the traditional Non-Maximum Suppression (NMS), facilitating an end-to-end training paradigm that forgoes NMS through a coherent dual-task assignment strategy, which in turn minimizes inference latency and expedites detection rates. The architecture of YOLOv10 is distinguished by its refined Backbone, Neck, and Head structures. The Backbone benefits from an advanced Cross Stage Partial Network that amplifies feature extraction prowess, while the Neck adeptly merges multi-scale features via the Path Aggregation Network layer. YOLOv10 introduces the pioneering One-to-Many Head to generate a spectrum of predictions during training, and the One-to-One Head to yield the most refined prediction during inference, all of which contribute to the model’s enhanced performance. In pursuit of superior mobile deployment, YOLOv10n has been designated as the foundational detection model for our endeavors.

#### 3.2.2 C2f-DCNv3

**Figure 3:**
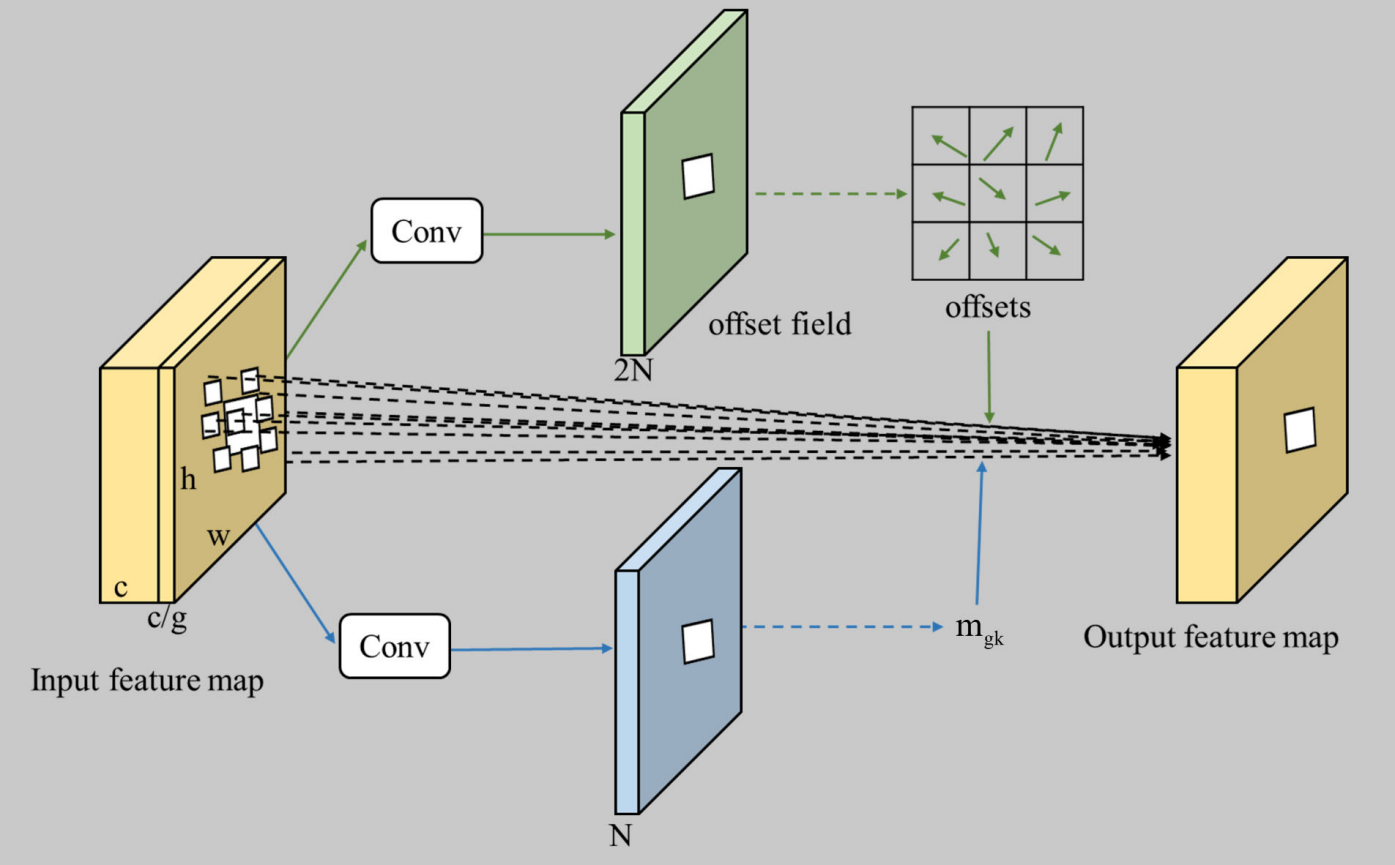
The structure of the DCNv3 module.


DCNv3 is a sophisticated convolutional core operator that enriches the standard convolutional process with the introduction of learnable offsets, enabling the kernels to adjust their sampling positions and conform to the intricacies of the input feature maps. This adaptive capability significantly improves the network’s ability to discern the contours and shapes of targets within an image (Wang et al., 2023). Evolving from its predecessors, DCNv3 has undergone substantial refinements, offering enhanced performance and efficiency (Zhu et al., 2019). The procedural flow of the DCNv3 module is illustrated in **Figure 3**. The input feature map is partitioned into g groups, each subjected to a convolutional operation to generate a corresponding set of offsets and modulation factors for the kernels. The final output feature map is then meticulously constructed from these predictive elements. The mathematical expression defining the deformable convolution v3 is articulated in Equation 1.

(1) $$y\left(p_{0}\right)=\sum_{g=1}^{G}\sum_{k=1}^{K}w_{g}m_{g}x_{g}\left(p_{0}+p_{k}+\Delta p_{gk}\right)$$

Where, $p_{0}$ is the pixel under consideration, G represents the number of groups, and K is the overall count of sampling points. The matrix $w_{g}$ is defined over *RC×C′*, where the group dimension is given by C′=C/G . The modulation scalar $m_{gk}$ for the k-th sampling point in the g-th group is subjected to normalization via a softmax function. The input feature map is denoted by $x_{g}$ in the space *RC×H×W*. The term $p_{k}$ corresponds to the k-th position sampled by the network, and $\Delta p_{gk}$ is the displacement related to the k-th grid sampling location.

**Figure 4:**
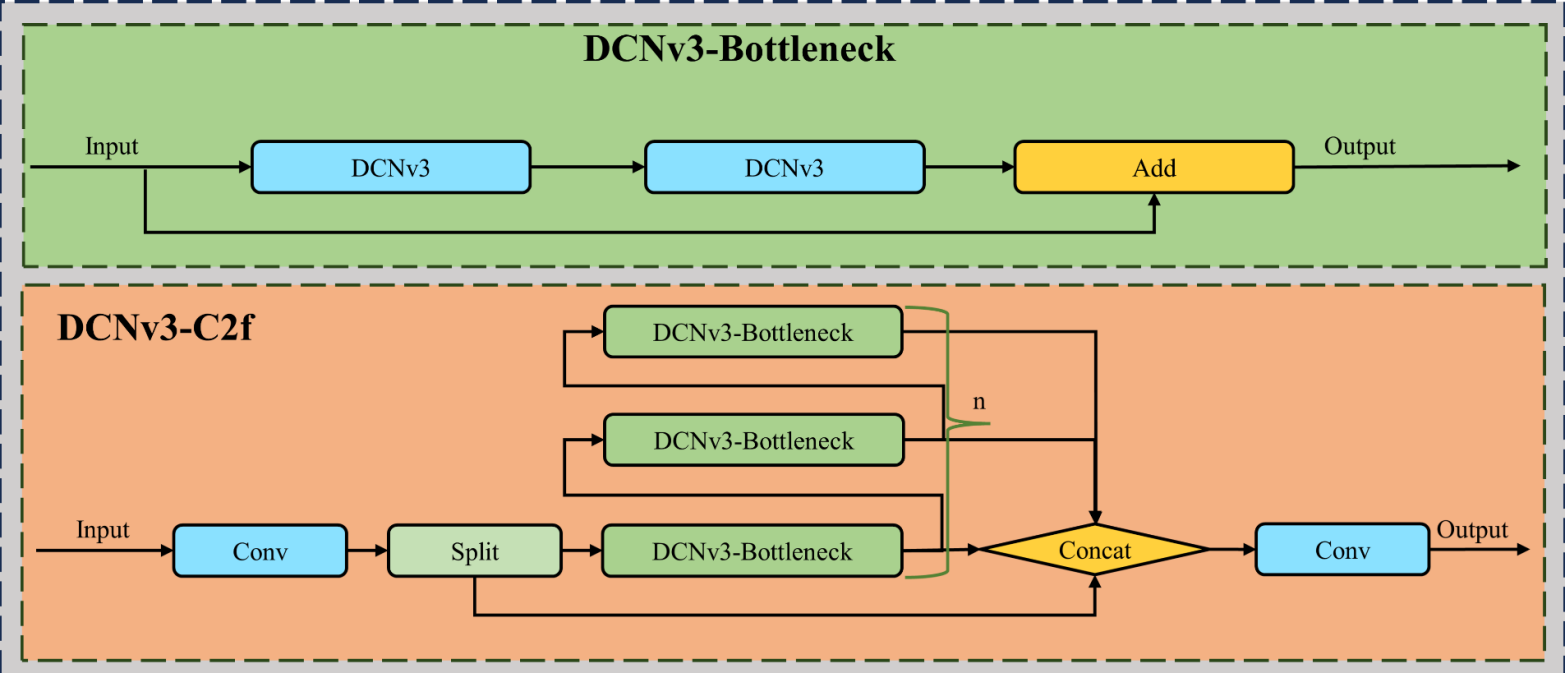
The structure of the C2f-DCNv3 module.

In this study, the DCNv3 module is employed to replace the convolutions within the C2f module, capturing spatial and channel information of the targets more effectively during the feature extraction phase, thereby enhancing the performance of the C2f module. The structure of the improved C2f-DCNv3 module is shown in **Figure 4**.

#### 3.2.3 Efficient local attention

The Efficient Local Attention (ELA) mechanism represents a cutting-edge innovation in attention mechanisms, crafted to escalate the efficacy and exactitude of feature extraction within the purview of deep learning models (Xu and Wan, 2024). Across the disciplines of Natural Language Processing and Computer Vision, attention mechanisms have become instrumental in advancing model capabilities. Despite the substantial computational demands and memory footprints of conventional global attention mechanisms, especially with extensive datasets, ELA offers a sophisticated solution. It harnesses self-attention on localized features, targeting discrete regions within the input feature maps, thereby substantially curtailing the computational and storage requisites.

**Figure 5:**
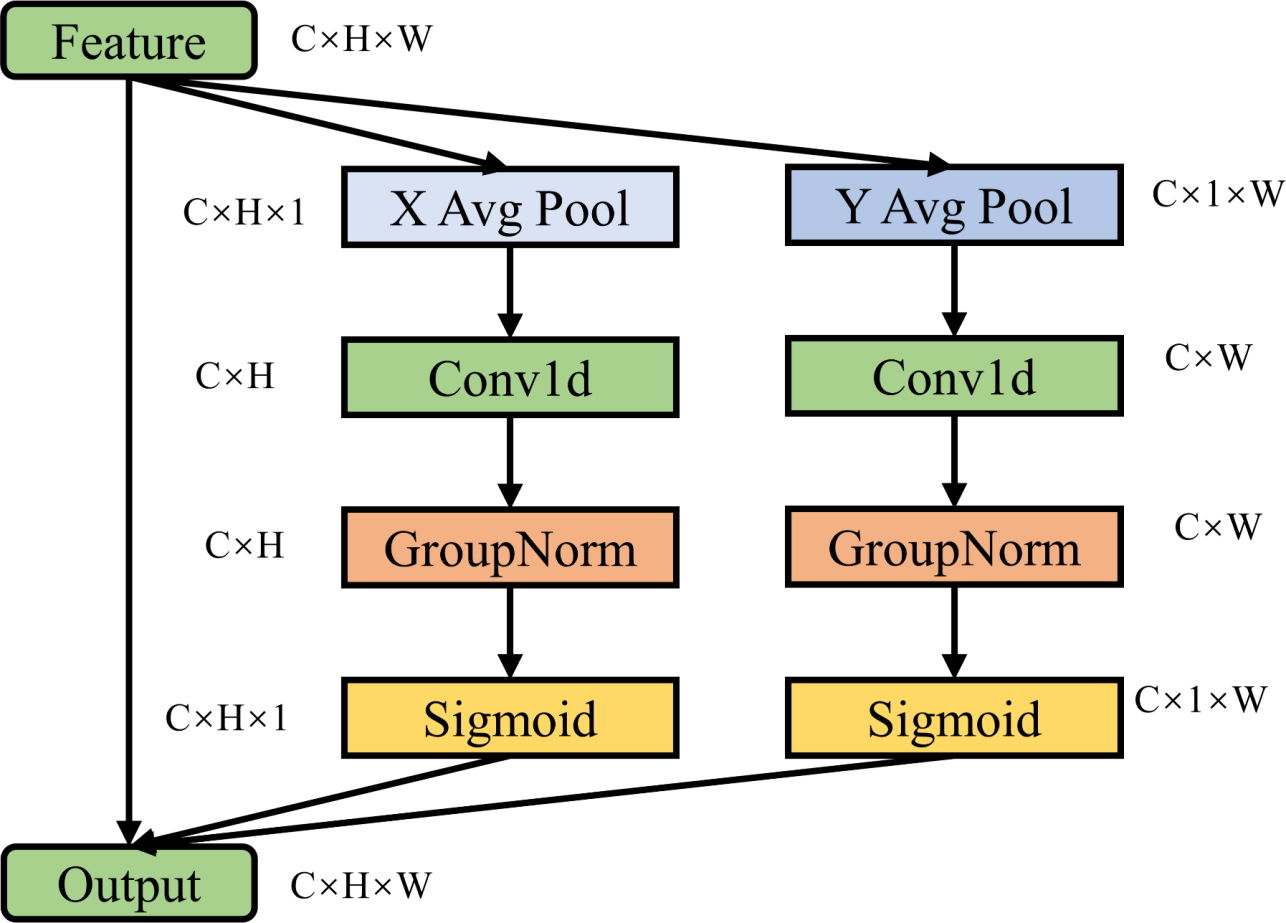
The structure of the ELA attention mechanism.

The essence of ELA’s superiority is its localized approach, as illustrated in **Figure 5**. By partitioning the input feature map into an array of compact windows and meticulously applying self-attention within the confines of each, ELA narrows its focus to local interactions, considerably attenuating the computational load. Moreover, ELA refines the computational expenditure by leveraging sparse sampling points to approximate the interrelatedness of local features, all without a detrimental impact on performance.

This research has implemented the ELA attention mechanism in place of the PSA attention mechanism within the YOLOv10n framework, aiming to bolster the model’s efficacy. Additionally, the integration of the ELA attention mechanism at the nexus of the backbone and neck network is intended to augment the model’s overall performance.

#### 3.2.4 Tobacco leaf detection network architecture

**Figure 6:**
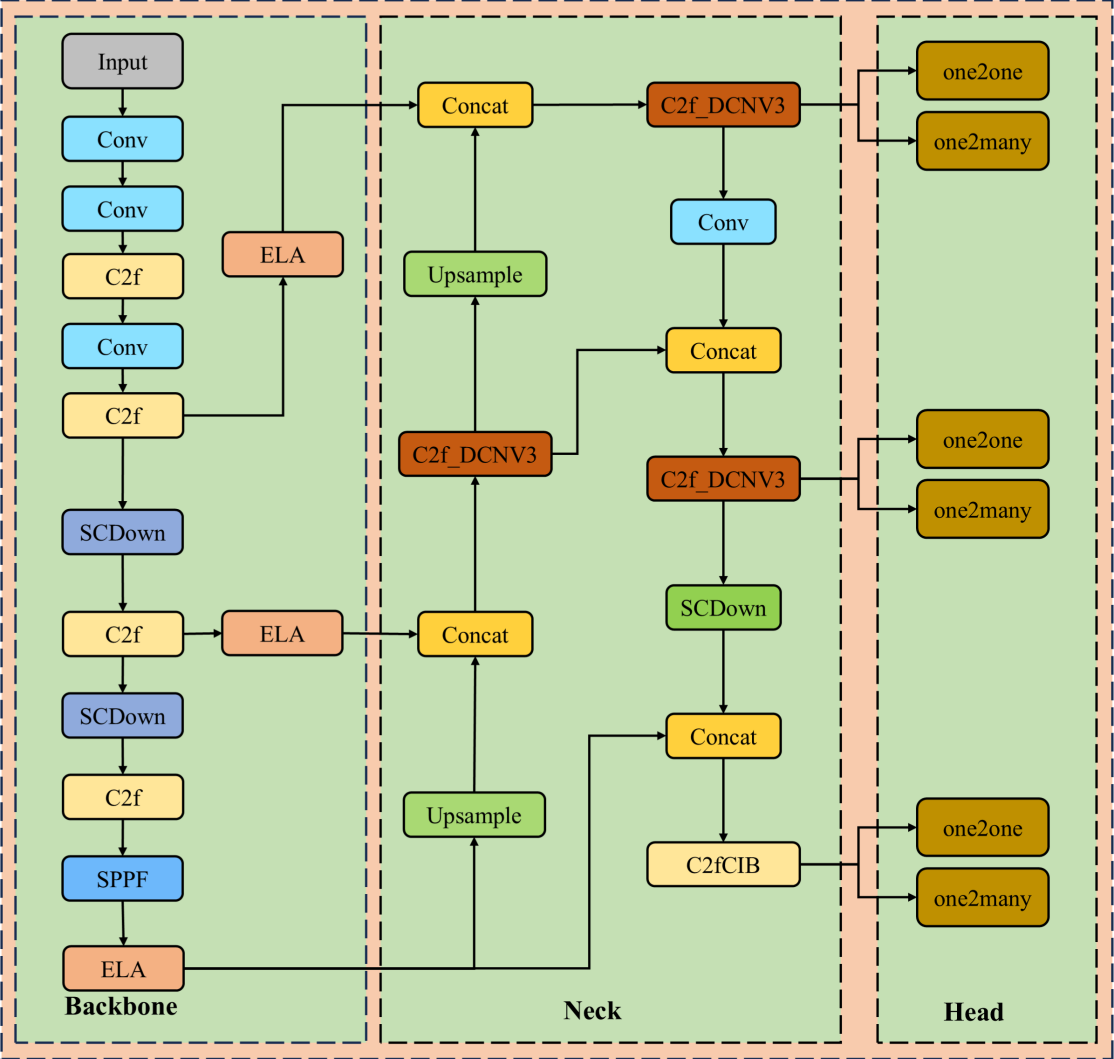
The structure of tobacco maturity network detection.

In this research, we have engineered a tobacco leaf maturity detection model predicated on the YOLOv10n framework. To amplify the model’s efficacy, we have innovatively combined the DCNv3 with the C2f module, resulting in an enhanced C2f_DCNv3 module. Moreover, we have introduced the ELA attention mechanism as a substitute for the PSA attention mechanism originally present in YOLOv10n. In addition to these modifications, we have strategically integrated the ELA attention mechanism at the interface between the backbone and the neck networks to further augment the model’s performance. The schematic representation of the tobacco leaf maturity detection network crafted in this study is illustrated in **Figure 6**.

### 3.3 Evaluation indicator

The present investigation applies Precision (P), Recall (R), mAP50, and mAP50-95 as the evaluative metrics for the tobacco leaf maturity detection model. Precision delineates the proportion of tobacco leaves that are accurately classified by the model into a specific maturity stage, signifying the model’s trustworthiness in predicting particular maturity levels. Recall measures the model’s effectiveness in identifying all instances of a given maturity stage, representing the ratio of correctly detected leaves to the total actual instances. mAP50 emerges as a holistic benchmark in the evaluation of tobacco leaf maturity, encapsulating the model’s aggregate proficiency in distinguishing among various stages. It is calculated by averaging the AP values across stages, thereby assessing the model’s comprehensive accuracy in classifying tobacco leaf maturity. mAP50-95 expands the IoU threshold scope, pivotal for nuanced visual feature differentiation across maturity stages. This metric furnishes an encompassing view of the model’s efficacy across a spectrum of matching stringencies. The respective computational formulas are articulated in Equations 2-5.


(2) $$\text{Precision}=\frac{TP}{TP+FP}$$

(3) $$\text{Recall}=\frac{TP}{TP+FN}$$

(4) $$mAP_{50}=\frac{1}{N}\sum_{i=1}^{N}AP_{i}$$

(5) $$mAP_{50-95}=\frac{1}{N}\sum_{i=1}^{N}\frac{1}{91}\sum_{j=1}^{91}AP_{i,j}$$

Where, TP is the tally of veracious positive instances, FP the tally of fallacious positive instances, and FN the tally of fallacious negative instances. *N* encapsulates the aggregate number of categories. $AP_{i}$ is the mean precision for the i-th category at an IoU threshold of precisely 0.5. $AP_{i,j}$ pertains to the mean precision for the i-th category at an IoU threshold incrementing from 0.5 by increments of 0.05 for each successive j, ranging up to 0.95. The term ‘91’ embodies the methodical computation of AP across this continuum, spaced into 91 uniform intervals for a meticulous assessment of AP.

## 4 Results and discussion

### 4.1 Experimental environment

The experimental procedures described herein were undertaken within a Windows 11 environment, leveraging the PyTorch deep learning framework at version 2.0.1, with Python 3.9 serving as the programming language of choice and PyCharm acting as the IDE for coding endeavors. The computational experiments were powered by an Intel Core i5-13500h CPU, complemented by 16 GB of system memory. The GPU designated for this research is the NVIDIA GeForce RTX 4050, endowed with 6 GB of graphics memory and 2560 CUDA cores for parallel processing capabilities. To ensure the reliability of our model, we adopted a consistent set of hyperparameters for all training runs. The hyperparameters for model training were sourced from https://github.com/THU-MIG/yolov10/blob/main/ultralytics/cfg/default.yaml. The specific values are summarized in the **Table 1**.

**Table 1:** Model training hyperparameter values.

Hyperparameter	Value	Description
lr0	0.01	Initial learning rate
lrf	0.01	Final learning rate (lr0 * lrf)
momentum	0.937	SGD momentum/Adam beta1
weight_decay	0.0005	Optimizer weight decay
warmup_epochs	3	Warmup epochs (fractions ok)
warmup_momentum	0.8	Warmup initial momentum
warmup_bias_lr	0.1	Warmup initial bias lr

### 4.2 Evaluation of the C2f-DCNv3 integration at distinct phases

In order to better evaluate the impact of C2f-DCNv3 on different parts of the model, this study utilizes C2f-DCNv3 to replace the C2f module in the backbone network and necking network, respectively, in order to enhance the performance of the model. The outcomes from integrating C2f-DCNv3 at these distinct phases are delineated in **Table 2**.

**Table 2:** The accuracy of the model for different stages of applying C2f-DCnv3.

Model		P	R	mAP50	mAP50-95
YOLOv10n	All	0.939	0.968	0.984	0.962
	Over-Mature	0.905	0.949	0.975	0.946
	Mature	0.923	0.966	0.984	0.972
	Immature	0.991	0.989	0.995	0.968
YOLOv10n+C2f-DCNv3(backbone)	All	0.97	0.974	0.991	0.962
	Over-Mature	0.952	0.96	0.989	0.956
	Mature	0.959	0.971	0.989	0.968
	Immature	0.999	0.991	0.995	0.961
YOLOv10n+C2f-DCNv3(head)	All	0.973	0.968	0.991	0.972
	Over-Mature	0.96	0.933	0.987	0.963
	Mature	0.967	0.972	0.991	0.979
	Immature	0.991	1	0.995	0.974
YOLOv10n+C2f-DCNv3	All	0.971	0.967	0.987	0.958
	Over-Mature	0.952	0.934	0.98	0.949
	Mature	0.962	0.966	0.987	0.969
	Immature	0.998	1	0.995	0.957

As indicated in **Table 2**, the overall model accuracy improved from 0.939 to 0.970, marking a 3.3% increase, when the C2f module in the backbone network was replaced in isolation. The mAP50 metric also saw a slight rise from 0.984 to 0.991, amounting to a 0.7% increase. Notably, within the “Immature” category, there was a significant leap in accuracy, with mAP50 and mAP50-95 experiencing boosts of 1.5% and 3.3%, respectively. Following the replacement of the neck network, the overall precision was further enhanced to 0.973, a 3.7% increase. The mAP50 metric mirrored the initial rise, while the mAP50-95 improved from 0.962 to 0.972, reflecting a 1.0% increase. Conversely, replacing the C2f modules in both the backbone and neck networks concurrently resulted in an overall precision of 0.971, yet the mAP50-95 dipped slightly to 0.958.

The incorporation of the C2f-DCNv3 module has notably enhanced the YOLOv10 model’s performance, particularly within the neck network structure. The C2f-DCNv3’s design amalgamates the profound feature extraction capabilities of Convolutional Neural Networks (CNNs) with the adaptability of Deformable Convolutional Networks (DCNs), thus enabling the model to adeptly adjust to the variability in target shapes and spatial configurations. Acting as a conduit between the backbone and detection head, the neck network’s efficacy is pivotal to the detection precision. Replacing the C2f module with C2f-DCNv3 in the neck network has bolstered the model’s target recognition by enriching feature representation. However, the decline in mAP50-95 when both networks are updated with C2f-DCNv3 could be attributed to potential issues. It may stem from overfitting due to heightened model complexity, especially with limited data. Alternatively, suboptimal feature integration strategies between the backbone and neck networks could lead to information loss or redundancy.

In this research, the strategy of replacing the C2f module in the neck network with C2f-DCNv3 has been selected from the outcomes of employing C2f-DCNv3 at various stages, as it demonstrated the most substantial benefit in enhancing model performance. Consequently, the C2f-DCNv3 module is chosen to replace the C2f module in the neck network to augment the model’s capabilities.

### 4.3 Model results with attention mechanisms added at different stages

In this research, we have made significant improvements to the YOLOv10n object detection model by incorporating the ELA (Efficient Layer-wise Attention) module to enhance the precision and efficiency of tobacco leaf maturation identification. Initially, we substituted the PSA (Pointwise Spatial Attention) mechanism in YOLOv10n with the ELA, creating the YOLOv10n+ELA1 model. Subsequently, we introduced an additional ELA module at the juncture of the backbone and neck networks within the YOLOv10n+ELA model to potentially elevate the model’s performance further. The precision of models with attention mechanisms modified at various stages is detailed in **Table 3**.

**Table 3:** Accuracy of the improved model for different stages of the attention mechanism.

Model		P	R	mAP50	mAP50-95
YOLOv10n	All	0.939	0.968	0.984	0.962
	Over-Mature	0.905	0.949	0.975	0.946
	Mature	0.923	0.966	0.984	0.972
	Immature	0.991	0.989	0.995	0.968
YOLOv10n+ELA1	All	0.964	0.971	0.986	0.965
	Over-Mature	0.95	0.967	0.983	0.958
	Mature	0.951	0.95	0.981	0.97
	Immature	0.991	0.998	0.995	0.966
YOLOv10n+ELA	All	0.972	0.97	0.992	0.966
	Over-Mature	0.95	0.96	0.989	0.96
	Mature	0.975	0.965	0.992	0.976
	Immature	0.991	0.986	0.995	0.961

From **Table 3**, it is clear that the enhanced model has shown significant performance improvements across all maturity categories. Specifically, for the “Over-Mature” category, the YOLOv10n+ELA1 model’s accuracy has increased from 0.905 to 0.95, and the mAP50 has improved from 0.975 to 0.983. In the “Immature” category, both accuracy and mAP50 have reached 0.991 and 0.995, respectively, demonstrating an exceptionally high recognition rate. Moreover, the YOLOv10n+ELA model has achieved an overall precision and mAP50 of 0.972 and 0.992 for the “All” categories, which is a 3.3% and 0.8% increase compared to the original YOLOv10n model.

The incorporation of the ELA module has notably bolstered the model’s capability to capture features indicative of tobacco leaf maturity. The ELA’s design, leveraging inter-layer attention mechanisms, effectively enhances the interconnectivity of feature maps, thus improving the model’s differentiation between tobacco leaves of varying maturities. Additionally, by incorporating ELA at the interface of the backbone and neck networks, we have further strengthened the conveyance and integration of features, enabling the model to sustain high recognition accuracy even when dealing with images of tobacco leaves against complex backgrounds and under diverse lighting conditions.

However, we have also noted a decrease in mAP50-95 for the “Over-Mature” category in the YOLOv10n+ELA1 model compared to the original model. This may indicate that the model’s ability to recognize extreme cases of tobacco leaf maturity has been somewhat compromised during the enhancement process. This could be attributed to the introduction of the attention mechanism, which may have altered the distribution of features, potentially diminishing the model’s generalization capabilities in certain scenarios.

### 4.4 Enhanced YOLOv10 model results through multi-stage fusion improvements

In this research, a comprehensive set of enhancements has been strategically applied to substantially elevate the performance of the YOLOv10 model. These improvements encompass the innovative replacement of the C2f module with the C2f-DCNv3 within the neck structure, alongside the sophisticated transition from the PSA (Pointwise Spatial Attention) mechanism to the ELA (Efficient Local Attention) mechanism within the backbone network. The seamless integration of an additional ELA attention mechanism at the interface of the backbone and neck networks has culminated in the development of a model that excels in the sophisticated recognition of tobacco leaf maturity. The model accuracy of the multi-stage improved fusion is shown in **Table 4**.

**Table 4:** Model accuracy for multi-stage improved fusion.

Model		P	R	mAP50	mAP50-95
YOLOv10n	All	0.939	0.968	0.984	0.962
	Over-Mature	0.905	0.949	0.975	0.946
	Mature	0.923	0.966	0.984	0.972
	Immature	0.991	0.989	0.995	0.968
ours	All	0.973	0.984	0.994	0.973
	Over-Mature	0.973	0.969	0.991	0.97
	Mature	0.97	0.992	0.995	0.981
	Immature	0.975	0.991	0.995	0.968

As demonstrated in **Table 4**, the enhanced model from this study surpasses the original YOLOv10n model in multiple indicators. In general, the precision (P) of our model across all categories has seen a rise from 0.939 to 0.973, which is a 3.4% increase; the recall (R) has also seen an improvement, increasing from 0.968 to 0.984, a 1.6% increase. The Mean Average Precision at 50% intersection over union (mAP50) has increased from 0.984 to 0.994, a 1.0% improvement; and the mAP50-95 has also shown an increase, moving from 0.962 to 0.973, a 1.1% increase.

In the granularity of specific categories, our model exhibits considerable improvement within the “Over-Mature” classification, with accuracy escalating from 0.905 to 0.973, reflecting a 6.8% enhancement; the recall rate has also witnessed an uptick from 0.949 to 0.969, a 2.0% gain; mAP50 has seen a boost from 0.975 to 0.991, a 1.6% advancement; and mAP50-95 has climbed from 0.946 to 0.970, a 2.4% escalation. Within the “Mature” classification, accuracy has surged from 0.923 to 0.970, amounting to a 4.7% enhancement; the recall rate has spiked from 0.966 to 0.992, a 2.6% augmentation; mAP50 has risen from 0.984 to 0.995, a 1.1% increment; and mAP50-95 has inched up from 0.972 to 0.981, a 0.9% increase. For the “Immature” classification, accuracy has slightly edged from 0.991 to 0.975; the recall rate has marginally improved from 0.989 to 0.991, a 0.2% increment; mAP50 has sustained its level at 0.995; and mAP50-95 has maintained its steadiness at 0.968.

The ELA demonstrates excellent performance in terms of computational efficiency and the enhancement of model capabilities. By adeptly capturing local features and providing advanced feature representation, ELA markedly boosts the model’s precision and generalization ability. Its primary strengths are the efficient capture of local features, optimization of channel dimensions, and a simplified structure, circumventing the redundancy and increased computational complexity inherent in global feature extraction. These attributes render ELA especially fitting for compact models and real-time applications, thus augmenting overall computational efficiency.

The C2f-DCNv3 module, a fusion of DCNv3 and the C2f module, strengthens the model’s adaptability to varied shape changes and spatial configurations. It leverages the adaptability of DCNv3 and the profound feature extraction capabilities of convolutional neural networks to further refine the model’s detection precision and robustness. The integration of the C2f-DCNv3 module into the neck network facilitates superior integration of multi-scale features, enhancing the accuracy of target recognition. Additionally, employing the ELA attention mechanism in conjunction with the C2f-DCNv3 module not only enhances detection precision but also bolsters the model’s robustness and generalization capabilities.

### 4.5 The results of the model comparison experiment

**Figure 7:**
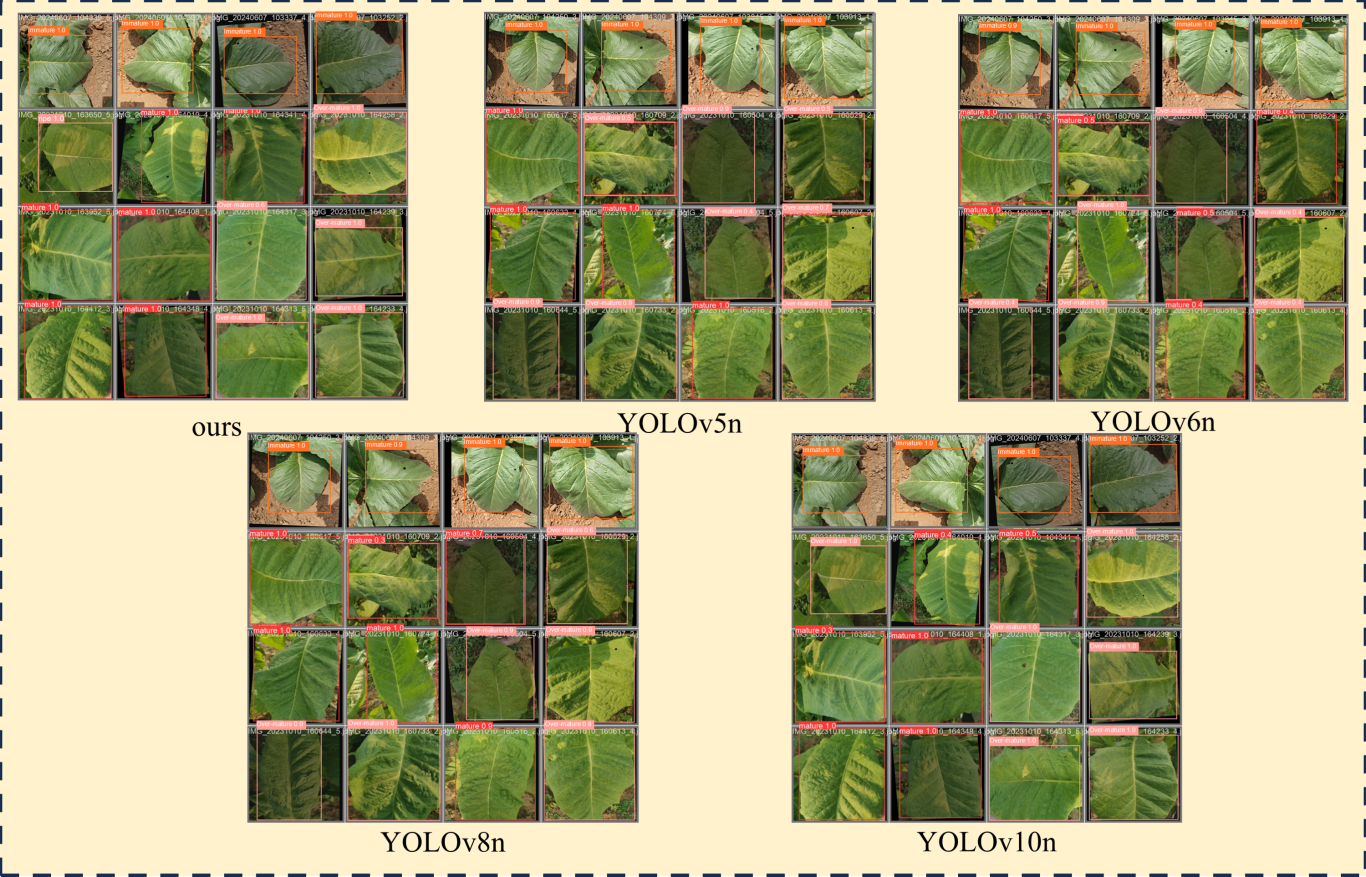
Tobacco maturity detection results.

To better demonstrate the capabilities of our model, this study compared it against four existing YOLO series models (specifically, YOLOv5n, YOLOv6n, YOLOv8n, and YOLOv10n). The comparative accuracy of these models is detailed in **Table 5**. The results of tobacco maturity detection for different models are shown in **Figure 7**.

**Table 5:** The experimental results of different models.

Model		P	R	mAP50	mAP50-95
YOLOv5n	All	0.919	0.966	0.983	0.933
	Over-Mature	0.871	0.948	0.978	0.932
	Mature	0.898	0.95	0.975	0.934
	Immature	0.988	1	0.995	0.934
YOLOv6n	All	0.928	0.909	0.962	0.931
	Over-Mature	0.904	0.867	0.942	0.92
	Mature	0.887	0.861	0.948	0.924
	Immature	0.993	1	0.995	0.95
YOLOv8n	All	0.949	0.948	0.984	0.951
	Over-Mature	0.951	0.903	0.978	0.945
	Mature	0.903	0.942	0.978	0.951
	Immature	0.992	1	0.995	0.958
YOLOv10n	All	0.939	0.968	0.984	0.962
	Over-Mature	0.905	0.949	0.975	0.946
	Mature	0.923	0.966	0.984	0.972
	Immature	0.991	0.989	0.995	0.968
ours	All	0.973	0.984	0.994	0.973
	Over-Mature	0.973	0.969	0.991	0.97
	Mature	0.97	0.992	0.995	0.981
	Immature	0.975	0.991	0.995	0.968

As depicted in **Table 5**, our model exhibited superior performance in the task of recognizing the maturity of tobacco leaves. Specifically, it achieved a P of 0.973, a R of 0.984, and mAP50 and mAP50-95 of 0.994 and 0.973, respectively, indicating exceptionally high detection accuracy. Within the subcategories representing different stages of maturity, our model continued to excel, maintaining mAP50-95 values above 0.968 for Over-Mature, Mature, and Immature categories, thereby highlighting the model’s high accuracy and robustness in identifying tobacco leaves at various stages of maturity.

In the horizontal analysis of the tobacco leaf maturity recognition models, our model demonstrated significant superiority across all four key performance metrics for all categories. For instance, when compared to the YOLOv10n model, our model showed improvements of 3.4% in precision, 1.6% in recall, 0.10% in mAP50, and 1.1% in mAP50-95. The performance gains were even more pronounced when compared to the YOLOv5n model, with increases of 5.4%, 1.8%, 1.1%, and 4.0% in these metrics, respectively. Similarly, when compared to the YOLOv6n model, our model’s improvements were 4.5% in precision, 7.5% in recall, 3.2% in mAP50, and 4.2% in mAP50-95. Although the YOLOv5n and YOLOv6n models showed good performance in certain metrics—YOLOv5n, for example, achieved an mAP50 of 0.995 for the immature category—our model overall exhibited a more outstanding comprehensive performance across all categories. While the YOLOv8n model was comparable to ours in some subcategories, such as a mAP50-95 of 0.945 for the Over-Mature category, our model showed higher consistency and stability across all maturity categories.

**Figure 8:**
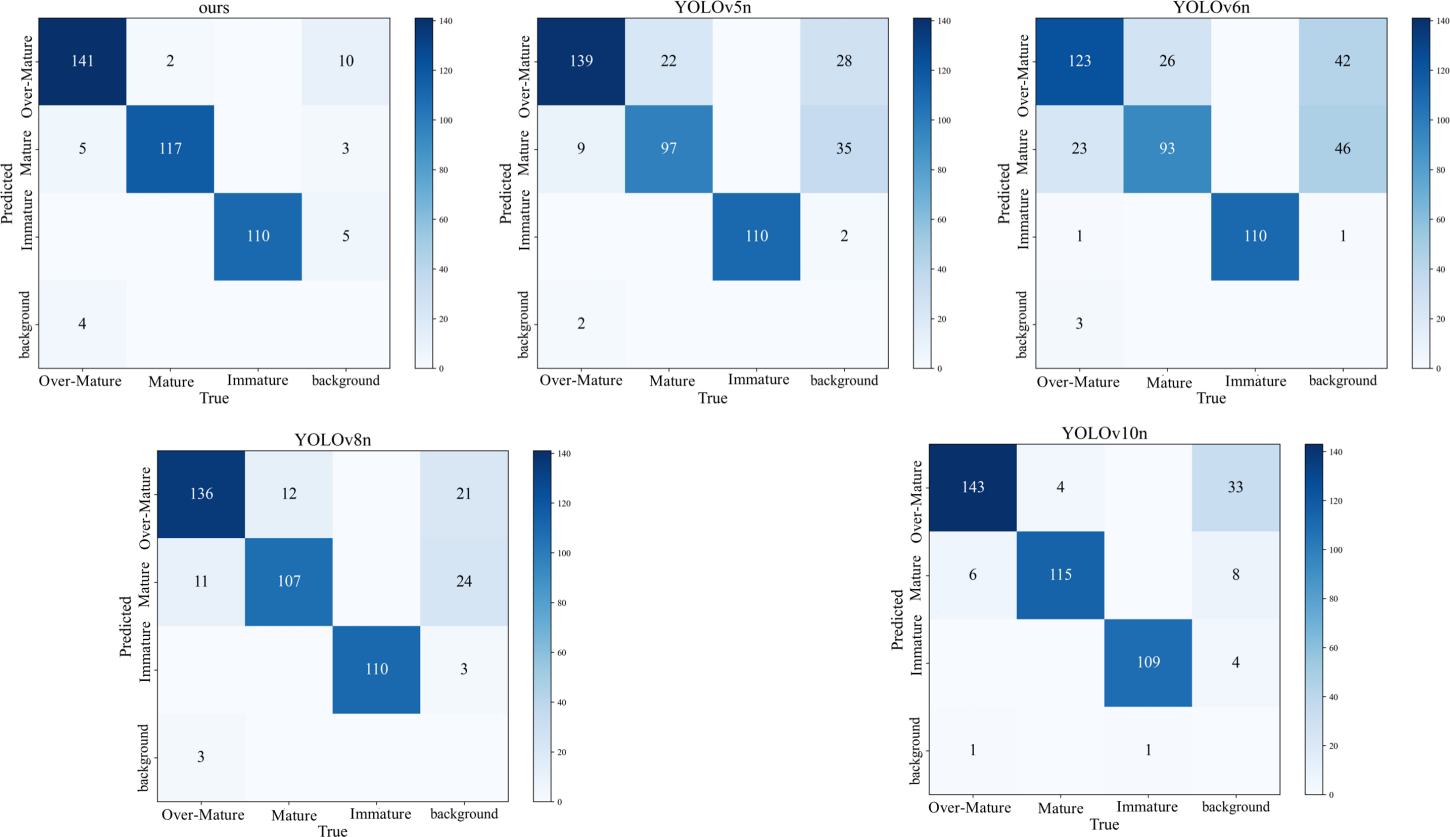
The confusion matrix of the detection results of different models.

To visually represent the performance of the models, a confusion matrix was employed to directly illustrate the detection capabilities. As shown in **Figure 8**, our model had the fewest misclassifications across the three maturity stages, followed by YOLOv10n, which had a lower total number of misclassifications. The YOLOv6n model performed the poorest, with the highest total number of misclassifications across all categories.

**Figure 9:**
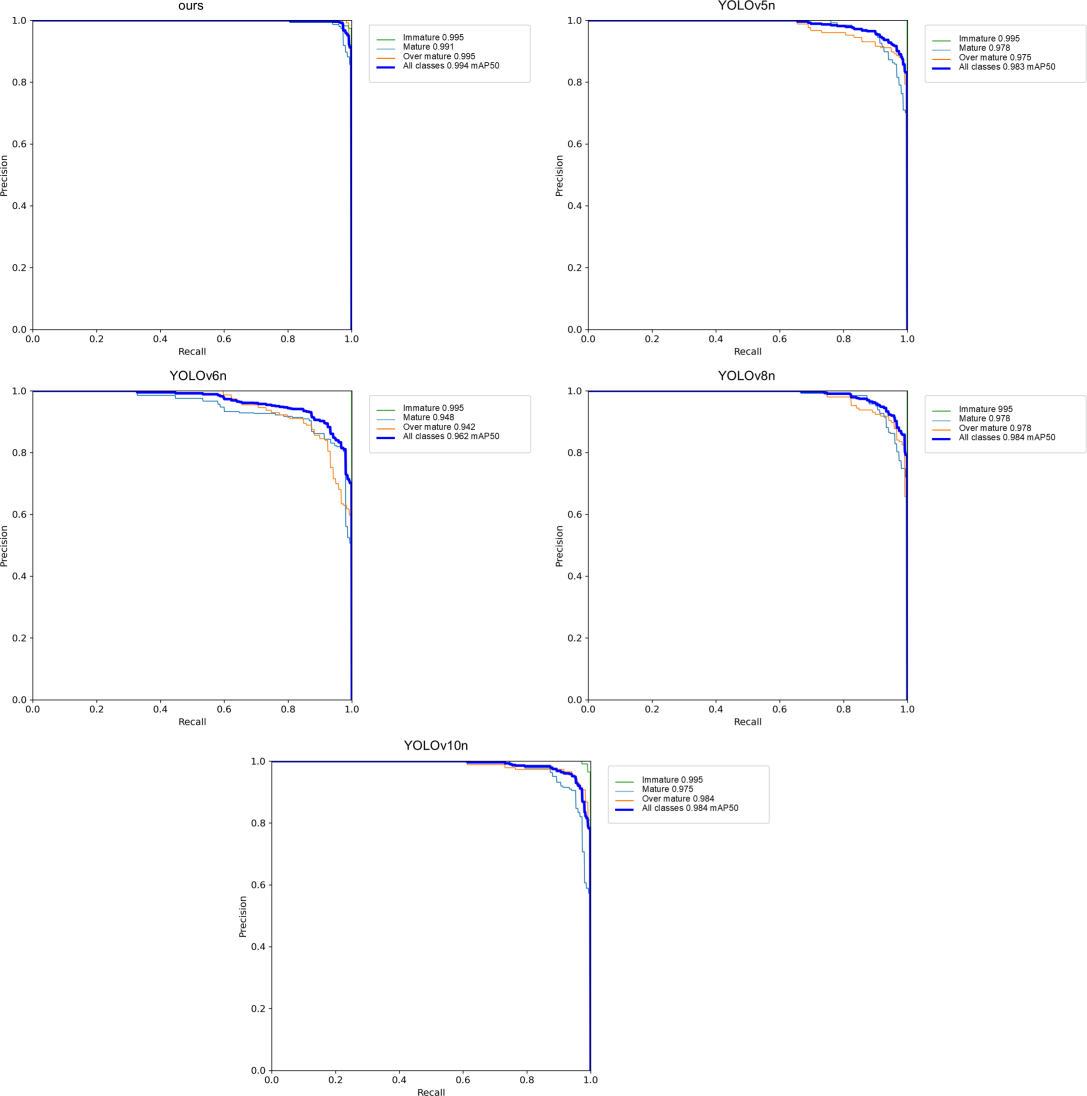
Precision-Recall graphs for different models.

To provide a more comprehensive evaluation of the model, this study employs the PR curve to assess the overall performance of the model in terms of recall and precision. The PR curves for different models are illustrated in **Figure 9**.

As shown in **Figure 9**, the ours model achieves a mAP50 of 0.994 at all categories, significantly surpassing other models. Specifically, YOLOv10n reaches a mAP50 of 0.984, while YOLOv5n and YOLOv8n achieve a mAP50 of 0.983 and 0.962, respectively. This indicates that the ours model has a distinct advantage in precision and recall, particularly maintaining a high level of precision in the high-recall region. Furthermore, the ours model also demonstrates outstanding performance in specific categories, achieving a mAP of 0.942 in the Over-Mature category, compared to YOLOv10n’s 0.975, suggesting that the ours model is slightly less effective in this category. However, in the immature and Over-Mature categories, the ours model achieves an mAP of 0.995 at a threshold of 0.5, showcasing its robust performance in these areas. Overall, the ours model exhibits excellent performance across multiple evaluation metrics, particularly with its overall performance of 0.994 mAP at 0.5, which is markedly higher than that of other models, underscoring its exceptional capabilities and potential in object detection tasks.

In conclusion, our model offers an efficient and precise solution in the domain of tobacco leaf maturity recognition. Its exceptional performance in key performance metrics, coupled with its clear advantages over existing models, underscores its significant potential for practical applications in agriculture. Future efforts will focus on further optimizing the model to minimize computational resource consumption and exploring its applicability in a broader range of agricultural monitoring tasks.

## 5 Conclusion

This research successfully developed a lightweight and efficient model for detecting the maturity of tobacco leaves by integrating DCNv3 to enhance the neck network of the YOLOv10 algorithm. We managed to optimize the model’s architecture without sacrificing detection precision, resulting in a reduction of parameter count and computational complexity. The experimental outcomes indicate that the application of the C2f-DCNv3 module in the backbone network elevated the overall precision from 0.939 to 0.970, and the mAP50 score from 0.984 to 0.991. Subsequent integration of the C2f-DCNv3 in the neck network achieved an overall precision of 0.973, with the mAP50 score sustained at 0.991, and a notable improvement in mAP50-95 from 0.962 to 0.972. Moreover, the incorporation of the ELA attention mechanism led to a significant boost in precision and mAP50 for the “Over-Mature” category, and an overall enhancement in the model’s performance across “All” categories, with accuracy and mAP50 scores increasing to 0.972 and 0.992, respectively. This study offers the tobacco industry a potent detection tool that can enhance the precision and efficiency of tobacco leaf harvesting, which is instrumental for improving tobacco leaf quality and the economic returns of tobacco farmers. Future endeavors will concentrate on further refining the model’s architecture to bolster its generalization capabilities and on investigating its practical application in field settings to ensure wider real-world utility and contribute to the sustainable growth of the tobacco industry.

## Data Availability

The raw data supporting the conclusions of this article will be made available by the authors, without undue reservation.
